# Abdominopelvic Tuberculosis with a Frozen Section Analysis Consistent with Ovarian Cancer

**DOI:** 10.1155/2017/6401694

**Published:** 2017-04-09

**Authors:** Agrimaldo Martins-Filho, Paula Carolina Arvelos Crispim, Renata Margarida Etchebehere, Cristina da Cunha Hueb Barata de Oliveira, Eddie Fernando Candido Murta, Rosekeila Simões Nomelini

**Affiliations:** ^1^Oncological Research Institute (IPON), Discipline of Gynecology and Obstetrics, Federal University of the Triângulo Mineiro, Uberaba, MG, Brazil; ^2^Surgical Pathology Service, Federal University of the Triângulo Mineiro, Uberaba, MG, Brazil; ^3^Infectious Diseases Service, Federal University of the Triângulo Mineiro, Uberaba, MG, Brazil

## Abstract

Pelvic tuberculosis is a type of extrapulmonary tuberculosis. The disease is accompanied by clinical and laboratory findings which may be unspecific and present aspects of other diseases, including gynecological malignancies. In this report, the authors presented a case of pelvic tuberculosis associated with peritoneal tuberculosis in a young woman exhibiting imaging and tumor markers consistent with ovarian neoplasm. An intraoperative frozen section analysis detected atypical cells that were suggestive of ovarian borderline or malignant epithelial neoplasia. The pathological analysis showed granulomatous inflammation in the right ovary and fallopian tube with a pattern of mycobacteriosis that was consistent with the presence of mycobacteria morphologically compatible with* Mycobacterium tuberculosis*. The patient had a complete remission after the use of antituberculosis drugs.

## 1. Introduction

Ovarian cancer cases are often characterized by the presence of ascites, adnexal masses, and high CA-125 levels. Radical surgery is generally the primary treatment, and this can include removal of the uterus, ovaries, fallopian tubes, and pelvic lymph nodes [1]. It has been reported that pelvic tuberculosis can be misdiagnosed as ovarian cancer based on the clinical and laboratory similarities between these two diseases. Moreover, pelvic tuberculosis can also mimic other pathological conditions including bowel disease, cancer, and infectious diseases [2].

Pelvic tuberculosis is a type of extrapulmonary tuberculosis that is caused by* Mycobacterium tuberculosis* [3, 4]. The symptoms for pelvic tuberculosis are generally nonspecific and often include a low-grade fever, abdominal pain, infertility, and menstrual disorders. The latter disease is rare in the Western world, while it is more common in nonindustrialized countries [5, 6]. However, due to greater opportunity for international travel and an increase in the incidence of immunosuppressive diseases such as AIDS, sporadic cases of peritoneal tuberculosis have been reported in greater number worldwide [1]. Risk factors include exposure to immigrants from countries with a high prevalence of the disease, urban poor, homeless, elderly individuals, and especially immunocompromised patients due to HIV infection [5, 7].

Here, we report a case of pelvic tuberculosis associated with peritoneal tuberculosis in a young woman exhibiting symptoms consistent with ovarian cancer.

## 2. Case Presentation

A 24-year-old woman presented to our Pelvic Mass Ambulatory Unit (Federal University of the Triângulo Mineiro (UFTM)) with a history of abdominal pain for the previous week that was associated with weight loss, fever, diarrhea, nausea, and ascites. A chest X-ray was normal. Abdominal ultrasonography showed an enlarged right ovary (3.2 × 4.0 × 4.0 cm; volume, 27.6 ml), with heterogeneous echogenicity, cystic areas, and thick walls. Magnetic resonance imaging (MRI) showed a heterogeneous mass with well-defined edges involving the right ovary that measured approximately 5.0 cm in diameter and exhibited heterogeneous contrast enhancement. The left ovary and uterus were normal. Additional observations included enlarged iliac lymph nodes, smaller lesions in the posterior fornix, abdominal ascites, and diffuse enhancement of the peritoneum. CA-125 and CA-15.3 levels were also increased (201 U/mL and 40.2 U/ml, resp.).

**Figure 1:**
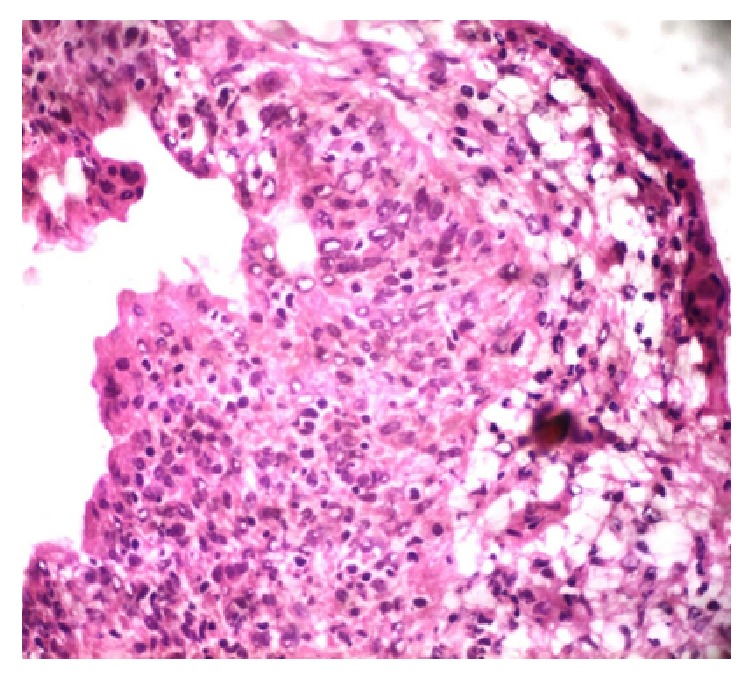
Intraoperative frozen section: atypical cells that were suggestive of ovarian borderline or malignant epithelial neoplasia.

An exploratory laparotomy was performed. The pelvis was frozen and the entire bowel and omentum were congested, similar to peritoneal carcinomatosis. During the procedure, biopsies were performed of the pelvic peritoneum, the fallopian tube, and the right ovary. An ascite sample was also collected. An intraoperative frozen section analysis detected atypical cells that were suggestive of ovarian borderline or malignant epithelial neoplasia (Figure 1). Despite these results, conservative surgery was performed based on the patient's desire to maintain her ability to become pregnant.

The pathological analysis showed granulomatous inflammation in the right ovary and fallopian tube with a pattern of mycobacteriosis that was consistent with the presence of mycobacteria morphologically compatible with* Mycobacterium tuberculosis*.

The patient was referred to the Infectious Diseases Service and received antituberculosis drugs. Six months later, the patient reported overall good health. Her CA-125 levels and an abdominopelvic ultrasound were both normal. Upon completion of the tuberculosis treatment, the patient was trying to become pregnant.

## 3. Discussion

Pelvic tuberculosis can present with nonspecific symptoms and similar laboratory and clinical findings to ovarian malignancy. Here, a patient with pelvic tuberculosis experienced pain and weight loss that were accompanied by increased tumor marker levels, the presence of ascites, and an ovarian mass. Surgery was necessary to confirm a diagnosis and treatment plan.

The presence of abdominopelvic pain, ascites, nausea, vomiting, and anorexia associated with imaging tests showing pelvic mass were reported in patients with abdominopelvic tuberculosis [4, 8–11] and may be associated with increased serum CA-125 levels [4, 8–10]. In these women, the association of these symptoms, imaging tests, and CA 125 levels mimicked ovarian cancer, delaying the tuberculosis diagnosis.

The treatment of pelvic tuberculosis is usually nonsurgical. It is difficult to diagnose pelvic tuberculosis prior to surgery, and currently there are no laboratory tests or imaging modalities that can differentiate this disease from advanced ovarian cancer [8]. Moreover, identification of a less invasive intervention for obtaining a differential diagnosis remains a major challenge. While a tuberculin skin test is an option, its low sensitivity may lead to false negatives in cases of abdominal tuberculosis [4, 12]. The present patient had no previous history of tuberculosis, her family history was negative, and she denied any contact with diseased individuals. Serological testing for sexually transmitted diseases was also negative. For a patient with a history of tuberculosis, close contact with infected individuals, travel to, or residency in, endemic countries, and immunodeficiency state are key considerations [13].

It is important to note that pelvic and pulmonary tuberculosis are not always associated. A standard chest X-ray is often performed, and computed tomography of the abdomen and pelvis can show ascites and induration of the mesentery and omentum [14]. CA 125 levels have been found to be increased in only half of patients with early epithelial ovarian tumors and in two-thirds of patients with advanced tumors. Since CA 125 levels can also be increased with pelvic tuberculosis, these levels are not very useful for a differential diagnosis of pelvic tuberculosis versus ovarian neoplasms [1]. However, in areas where tuberculosis is endemic, the detection of high levels of CA-125 in premenopausal women should lead to further examination of a tuberculosis diagnosis [8]. In the present case, the levels of CA-125 and CA-15.3 were increased and the chest X-ray was normal.

A BAAR analysis of ascites also has a low sensitivity for a diagnosis of peritoneal tuberculosis. However, an advance in the analysis of ascitic fluid in this context has been the detection of adenosine deaminase (ADA). This enzyme is involved in the conversion of adenosine to inosine and is released by macrophages and lymphocytes during an immune response. In a previous study it was demonstrated that a cutoff level of 39 U/I for ADA levels provided high sensitivity and specificity for a diagnosis of abdominopelvic tuberculosis [15]. Further studies are needed to confirm the use and sensitivity of this assay.

Laparoscopy as a diagnostic tool in combination with biopsy and histology was found to be effective for establishing a diagnosis of pelvic tuberculosis. Laparoscopic biopsy is a sensitive and specific diagnostic procedure for abdominal peritonitis, and it is considered to be the gold standard method for clearly differentiating advanced ovarian cancer and peritoneal tuberculosis, especially in areas where tuberculosis is endemic [16]. However, the use of laparoscopic surgery has also been associated with higher complication rates in patients with pelvic tuberculosis, and laparoscopic findings can resemble those of metastatic ovarian cancer. Therefore, only a biopsy that shows caseating granulomas in a histopathological analysis can provide a definitive diagnosis of tuberculosis. Meanwhile, intraoperative frozen section analyses that reveal caseous necrosis with granulomatous lesions can potentially prevent unnecessary oophorectomies in young women [17, 18]. In the present case, cellular atypia in the frozen sections indicated ovarian cancer.

Although frozen section has good accuracy for malignant and benign neoplasms, its efficiency in detecting borderline ovarian tumors is not high, perhaps because more extensive samples are needed [19, 20]. Intraoperative frozen section sensitivity ranges from 86.1% to 92.5%, 98.2% to 100%, and 44.8% to 87% in malignant, benign, and borderline tumors, respectively. Its specificity ranges from 98.5 to 100%, 87 to 92.8%, and 96.4 to 98.6%, respectively [19–23]. Thus, conservative surgeries should be performed when intraoperative frozen section shows borderline tumor or malignant tumor in young patients wishing to preserve fertility.

The ability to provide a preoperative differential diagnosis of pelvic tuberculosis that mimics an ovarian tumor remains a challenge, especially in the absence of specific protocols, nonspecific symptoms, and the rarity of cases that characterize this disease. As demonstrated in the present case, conservative surgery should be performed when a diagnosis is uncertain, especially for patients who wish to become pregnant.
